# *Actinomyces odontolyticus* lung abscess and pleural empyema

**DOI:** 10.34172/aim.2022.65

**Published:** 2022-06-01

**Authors:** Hang Ruan, Yi-ming Tao, Shu-sheng Li

**Affiliations:** ^1^Department of Critical-care Medicine, Tongji Hospital, Tongji Medical College, Huazhong University of Science and Technology, Wuhan, 430030, China

**Figure 1:**
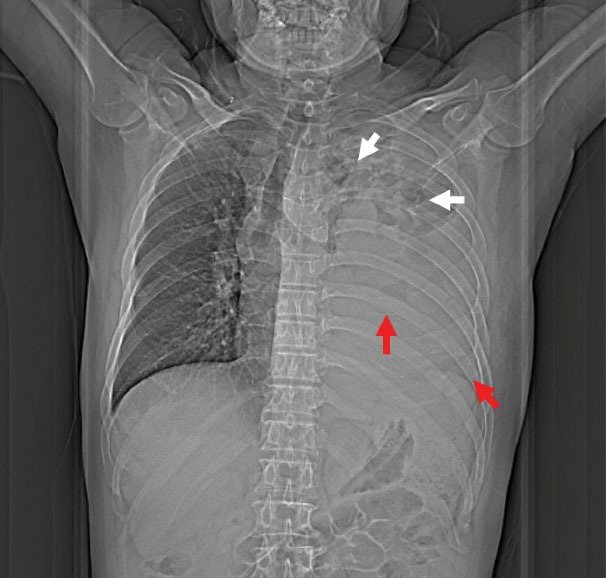
Chest X-ray at Admission Demonstrating Multiple Cavitary Lesions in Upper Left Zone (White Arrow) and Massive Pleural Effusion in the Left Lower Thorax (Red Arrow).

**Figure 2:**
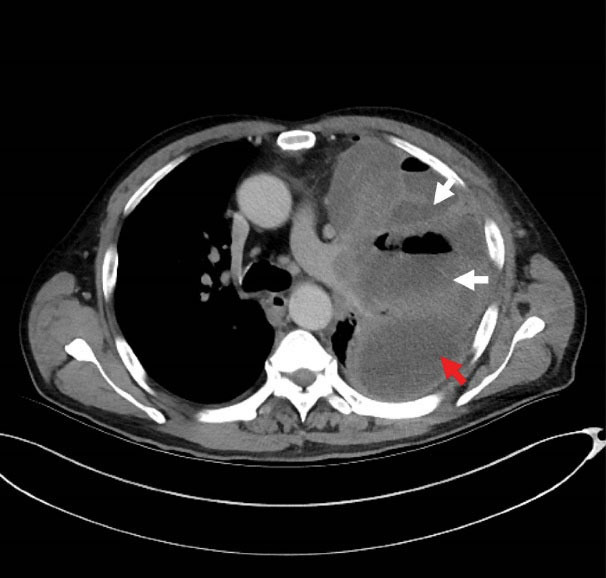
Chest Computed Tomography Showing Multiple Pulmonary Abscesses Formation (White Arrow) and Pleural Empyema (Red Arrow).

**Figure 3:**
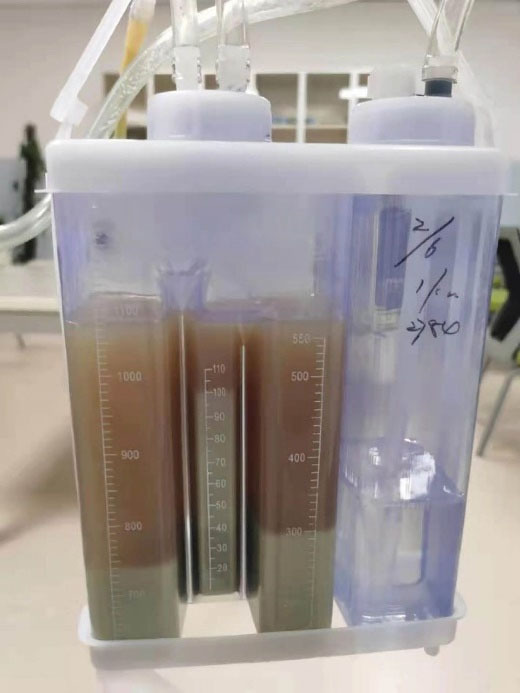
Gross Appearance of Pleural Effusion.

**Figure 4:**
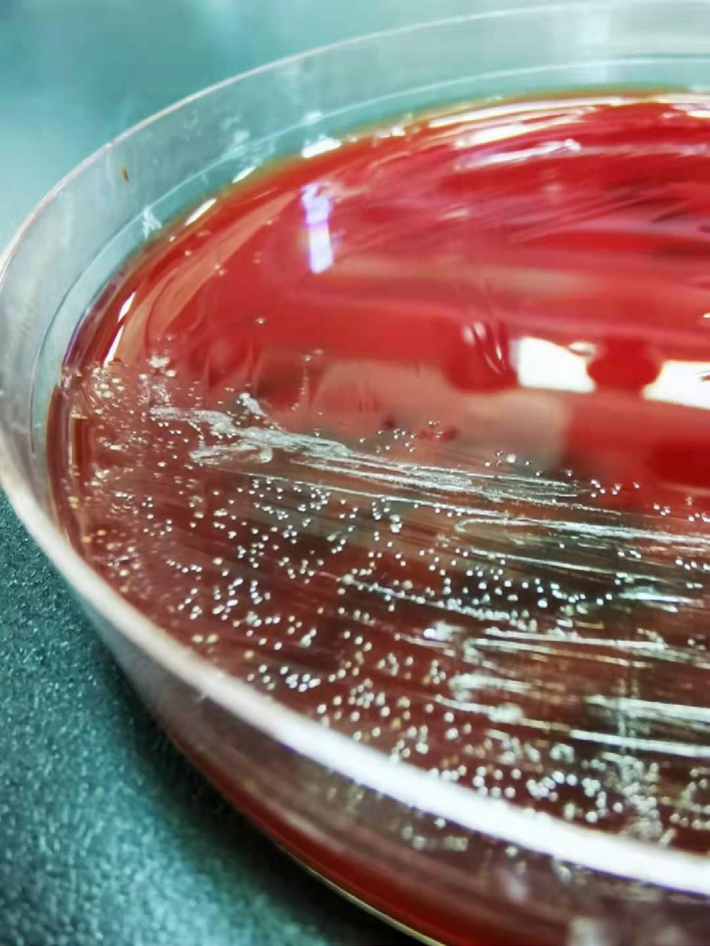
Confirmation of Actinomyces Odontolyticus Infection by Microbiology Culture.

**Figure 5:**
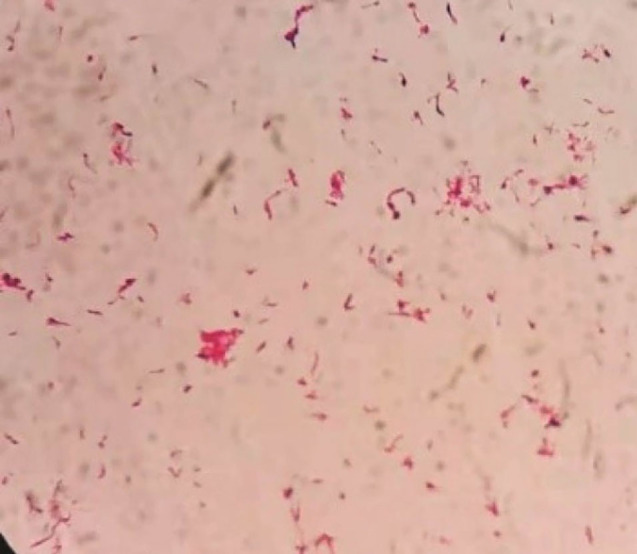
Light Microscope Image of Bacteria (× 1000).

 A 63-year-old patient with left chest pain was admitted to the emergency department for ten days. His clinical symptoms had rapidly progressed to persistent pain, abundant purulent sputum, and severe hypoxemia at the time of admission. He also had a 40-pack-per-year history of smoking with poor oral hygiene. Physical examination revealed that the patient had decreased respiratory sounds in the left hemithorax, with dullness on percussion. Laboratory findings revealed that the patient’s white blood cell count was 16.3 × 10^9^/L, and his hypersensitive C-reactive protein (hs-CRP) was 110 mg/dL (N < 10). Chest radiographs indicated multiple cavitary lung lesions with massive pleural effusion (Figure 1). Chest CT scan further confirmed multiple pulmonary abscess and empyema (Figure 2). Afterward, a thoracic drainage tube was placed, and more than 1000 mL of purulent fluid was drained (Figure 3). Purulent fluid culture determined the presence of *Actinomyces odontolyticus* (Figures 4 and 5). Unfortunately, despite high-dose penicillin therapy and chest tube drainage, the patient succumbed to respiratory failure caused by rapid disease progression in *A. odontolyticus* infection one week later.

 Actinomycetes are prokaryotic organisms with both gram-positive bacterial and fungal properties. They cause various chronic pyogenic granuloma diseases collectively known as actinomycosis, occurring on the neck, face and thoracic abdomen and progressing slowly, characterized by fistulae and yellowish sulfur granules.^1^ The common pathogenic species include *Actinomyces israelii*, *Actinomyces naeslundii*, *Actinomyces viscous*, and *Actinomyces odontolyticus*. *A. israelii* infection is the most prevalent, while *A. odontolyticus* infection is the most infrequent.

 Actinomycosis may be an invasive bacterial infection and is associated with various etiologies. Dental caries has been recognized as a high-risk factor for actinomycosis. The aspiration of oropharyngeal or gastrointestinal secretions is considered another cause of pulmonary actinomycosis.^2^ Next, blood-borne dissemination caused by the destruction of the integrity of the oral mucosa barrier has been rarely reported.^3^

 The imaging features of actinomycosis cases with respiratory involvement include: consolidation, mediastinal and hilar lymphadenopathy, atelectasis, cavitation, and ground-glass shadows.^4^ In addition, pleuropulmonary infections are rare. Relevant treatment options include medication and chest tube drainage, while the preferred drug regimen is high-dose prolonged penicillin therapy. Metronidazole, fluoroquinolones, and aminoglycosides show no activity against actinomycetes *in vitro*. Meanwhile, in case of penicillin allergy, macrolides and clindamycin may be alternatives.^5^
